# Non-toxic dose of liposomal honokiol suppresses metastasis of hepatocellular carcinoma through destabilizing EGFR and inhibiting the downstream pathways

**DOI:** 10.18632/oncotarget.13687

**Published:** 2016-11-29

**Authors:** Jianhong Yang, Heying Pei, Hong Luo, Afu Fu, Hansuo Yang, Jia Hu, Chengjian Zhao, LuLu Chai, Xiang Chen, Ximing Shao, Chunyu Wang, Wenshuang Wu, Li Wan, Haoyu Ye, Qiang Qiu, Aihua Peng, Yuquan Wei, Li Yang, Lijuan Chen

**Affiliations:** ^1^ State Key Laboratory of Biotherapy and Cancer Center, West China Hospital, Sichuan University, and Collaborative Innovation Center for Biotherapy, Chengdu, P.R. China; ^2^ Department of Ultrasonic Medicine, West China Second Hospital, Sichuan University, Chengdu, China; ^3^ School of Pharmacy, Chengdu University of TCM, The Ministry of Education Key Laboratory of Standardization of Chinese Herbal Medicine, State Key Laboratory Breeding Base of Systematic Research, Development and Utilization of Chinese Medicine Resources, Chengdu, China

**Keywords:** liposomal honokiol, EGFR, metastasis, motility, hepatocellular carcinoma

## Abstract

At present, there is no specific anti-metastasis drug in HCC treatment. Drugs used for primary HCC tumors and tumor metastasis are very similar, among which cytotoxic drugs are prevalent, such as cisplatin, doxorubicin and 5-FU. The EGFR pathway plays an important role in promoting hepatocellular carcinoma (HCC) metastasis. Hence, development of non-toxic anti-metastasis drugs, such as EGFR or downstream pathways inhibitors, is of great importance. In our present study, we found non-toxic dose of liposomal honokiol (LH) could inhibit the HCC metastasis by destabilizing EGFR and inhibiting the downstream pathways. Non-toxic dose of LH significantly inhibited the motility, migration and lamellipodia formation of HepG2 cells *in vitro* and decreased extravasation of HepG2 cells in a novel metastasis model of transgenic zebrafish. In two lung metastasis models (HepG2 and B16F10) and a spontaneous metastasis model of HepG2 cells, LH remarkably inhibited pulmonary metastasis and regional lymph nodes metastasis without obvious toxicity. Further study showed that destabilizing EGFR and inhibiting the downstream pathways were the main mechanisms of non-toxic dose of LH on metastasis inhibition. Our results provide the preclinical rationale and the underlying mechanisms of LH to suppress HCC metastasis, implicating LH as a potential therapeutic agent to block HCC metastasis without severe side effects.

## INTRODUCTION

Hepatocellular carcinoma (HCC) is one of the most common malignant tumors characterized by rapid progression, easy metastasis, and frequent recurrence [1–5]. It is the second leading cause of cancer mortality in China [4]. HCC frequently shows early invasion into blood vessels as well as intrahepatic metastasis, and later demonstrates extrahepatic metastasis. Extrahepatic metastasis, most frequently located in the lung and the regional lymph nodes, is a major cause of the poor prognosis and HCC-related death [1–5].

The metastasis process normally compromises migrating and invading of tumor cells into extracellular matrix of surrounding tissues, entering and circulating in host vasculatures, extravasating, and establishing metastatic colonies in second sites [6]. In theory, inhibition of any of the steps in the metastatic process could offer therapeutic targets [2, 4, 6]. However, there are no specific drugs used for anti-metastasis treatments in clinical chemotherapy [7]. The drugs used for primary tumors and tumor metastasis are very similar in HCC treatment, among which cytotoxic drugs are prevalent, such as cisplatin, doxorubicin and 5-FU [8, 9]. Unfortunately, these drugs also display cytotoxicity to normal cells resulting in severe side effects to patients such as anaemia, nausea, vomiting, weight loss, fatigue, and increased chance of infections. Considering the distinct biological characteristics between tumor metastasis and primary tumors [6], we hypothesize that a non-toxic candidate drug might be effective to suppress the metastasis of hepatocellular carcinoma without causing severe side effects.

The EGF-EGFR pathway plays an important role in cancer metastasis by activating the downstream PI3K/Akt, ERK and JNK pathway [10], the inhibition of EGFR or its downstream signal pathway will inhibit cancer metastasis, which sometimes will not reduce cell viability [11]. Hence, non-toxic EGFR or downstream pathway inhibitors may be effective anti-metastasis drugs in HCC without causing obvious side effects.

Honokiol is an active component isolated from the root and stem bark of magnolia, a plant used for centuries in traditional Chinese and Japanese medicine. Honokiol is known to possess anti-thrombocytic, antibacterial, anti-inflammatory, antiproliferative and anxiolytic effects [12–16]. Studies have demonstrated antineoplastic and anti-angiogenic properties of honokiol for mouse or human cancer cells, including lung cancer, leukemia, multiple myeloma, colon cancer, prostate cancer, fibrosarcoma and ovarian carcinoma [17–27]. Our previous studies have shown that honokiol possesses significant antitumor effect as well as an enhancement in tumor growth delay and improvement of survival by combination with cisplatin in ovarian carcinoma [27] or with adriamycin in breast cancer models [28]. The poor water solubility of honokiol could be improved by encapsulating honokiol with PEG modified liposome for *in vivo* i.v administration. Further studies identified that liposomal honokiol (LH) inhibits VEGF-D-induced lymphangiogenesis and metastasis in xenograft tumor model [29], However, it is still unclear whether non-toxic dose of LH could inhibit the early invasion and intrahepatic metastasis of HCC and the underlying mechanism has not been fully investigated.

## RESULTS

### Non-cytotoxic LH reduces hepatocellular carcinoma cells motility and inhibits cells migration

**Figure 1:**
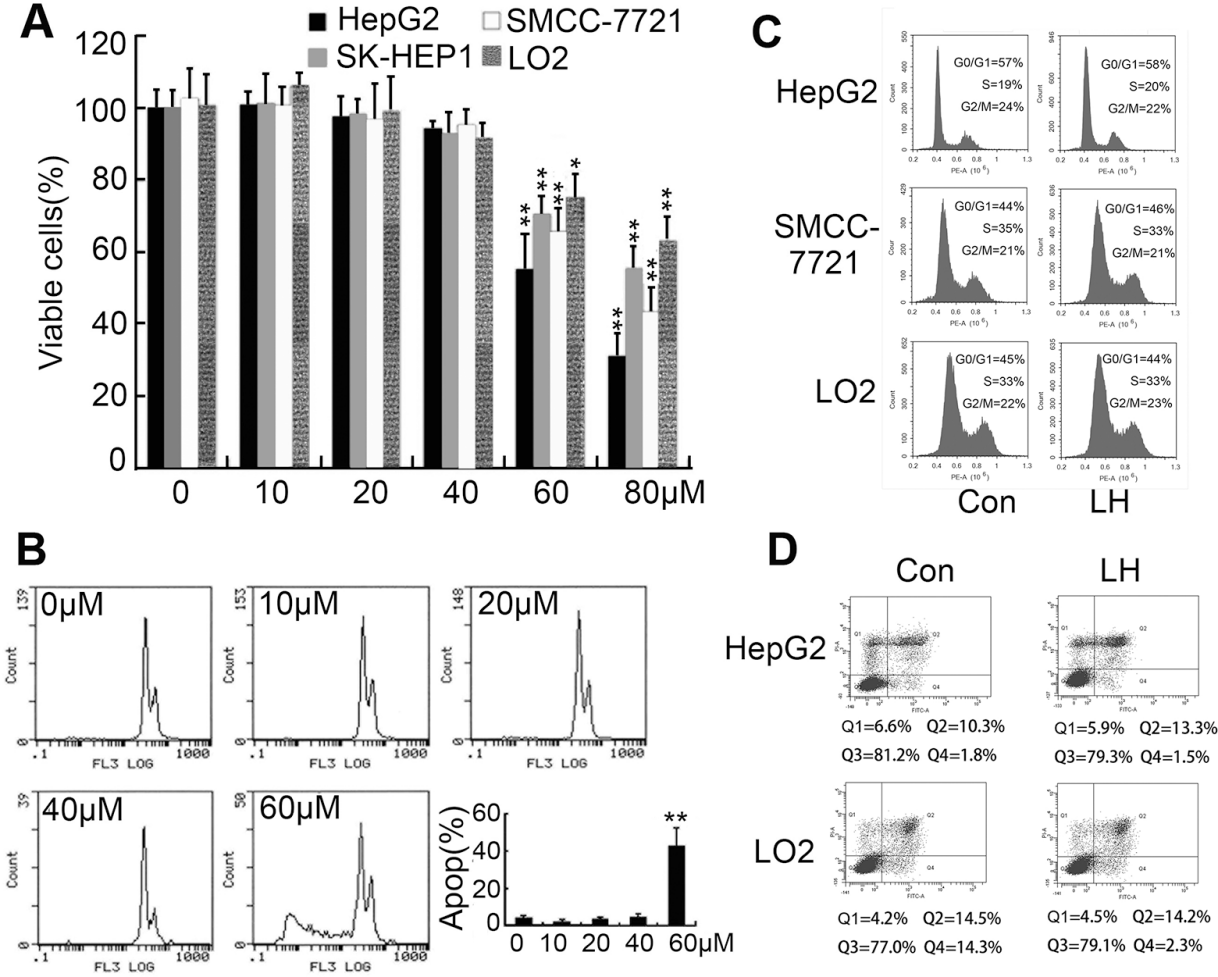
Determination of non-toxic concentration of LH (**A**) The inhibitory effect of LH on HepG2, SM7721, SK-HEP1 and LO2 cell proliferation. The inhibition of cell proliferation was determined using MTT assay. The cells were treated with empty liposome or the indicated concentrations of LH for 24 h. Data represent the mean ± standard error (SE) from three independent experiments. *P < 0.05, **P < 0.01, compared with the empty liposome group. (**B**) HepG2 Cells were treated with empty liposome or different concentrations of LH for 24 h, then collected, stained with PI, and analyzed by flow cytometry for apoptosis. Data represent the mean ± SE from three independent experiments. **P < 0.01, compared with the empty liposome group. (**C**) Cells (HepG2, SMCC7721 and LO2) were treated with empty liposome or 40 μM LH for 24 h, then collected, stained with PI, and analyzed by flow cytometry for cell cycle. (**D**) Cells (HepG2 and LO2) were treated with empty liposome or 40 μM LH for 24 h, then collected, stained with PI and AnnexinV, and analyzed by flow cytometry for apoptosis.

We firstly intended to identify whether nontoxic dose of liposomal honokiol could reduce hepatocellular carcinoma cells motility and inhibit cells migration. The liposomal honokiol was prepared in our laboratory [29]. There is no difference between liposomal honokiol and free honokiol in antiproliferative activity in tumor cells [30]. *Rajendran et al* showed 25 μM honokiol treatment for 24 h could reduce HepG2 cell viability [31]. Recently, Min *et al* showed higher dose of honokiol (up to 100 μM) treatment for 24 h showed no obvious effect on cell viability of HepG2 cells [32]. With these contradictory results, we first evaluated the cytotoxicity of LH on HCC cell lines (HepG2, SK-HEP1 and SMCC7721) and normal liver cells (LO2) in a series of concentration by MTT. We found that LH showed cytotoxicity on tested cell above concentration of 60 μM after treatment for 24 h, but no cytotoxicity under 40 μM concentration (Figure 1A). Then HepG2 cells treated with indicated concentration of LH were subjected to PI staining for apoptosis analysis, we found that 60 μM LH induced obvious apoptosis but 40 μM or lower concentration induced no apoptosis (Figure 1B). Furthermore, PI staining of HepG2, SMCC7721 and LO2 cells treated with 40 μM honokiol were subjected to flow cytometry for cell cycle analysis. Results demonstrated that LH at 40 μM did not cause obviously cell cycle arrest on these cells (Figure 1C). Furthermore, PI/AnnexinV stain for apoptosis analysis further confirmed that 40 μM LH caused no obvious apoptosis on HepG2 and LO2 cells (Figure 1D). Hence, we demonstrated that 40 μM LH is a non-toxic concentration on HCC cells.

**Figure 2:**
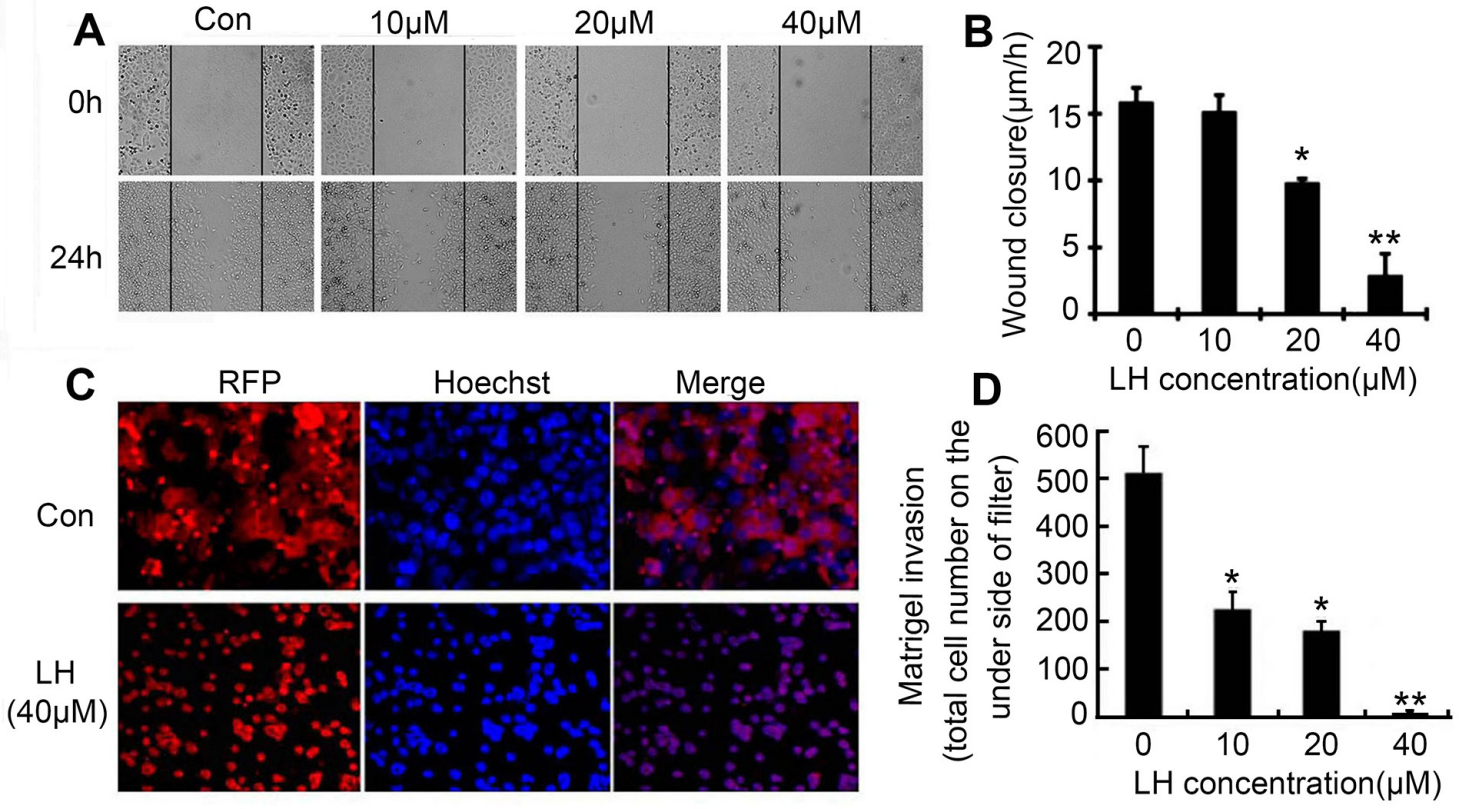
LH inhibited migration and invasion of HCC cells at non-toxic concentrations (**A**) The effect of LH at indicated concentration on HepG2 cells migration in wound-healing assays. Cells were scratched with a pipette tip and then treated with empty liposome or indicated concentrations of LH for 24 h. Migrating cells were photographed under a phase contrast micoscopy. The data are representative of 3 independent experiments (**B**) Migrating cells per fields in wound-healing assays were evaluated after treated with indicated concentration of LH. Data represent the mean ± standard error (SE) from three independent experiments. *P < 0.05, **P < 0.01, compared with the empty liposome group (**C** and **D**) HepG2 cells transfected with red fluorescent genes (pDsRed-N1) were treated with empty liposome or LH (10, 20, 40 μM) for 24 h, and the invasive ability was assessed by a matrigel-coated transwell assay, the total cell number on the underside of filter were counted. The data are representative of 3 independent experiments. Data are expressed as mean ± SE. *P < 0.05, **P < 0.01, compared with the empty liposome group.

Since LH at 40 μM did not cause obvious apoptosis, we further investigated whether LH could inhibit the cells migration and invasion at non-cytotoxic concentrations (≤ 40 μM) by wound-healing migration and transwell cell invasion assays. Hence, we choose non-toxic concentrations (≤ 40 μM) of LH to evaluate the ability to inhibit the migration and invasion in HepG2. As shown in Figures 2A and 2B, HepG2 cells obviously migrated to the wound after 24 h exposure, by contrast, treatment with LH at non-toxic concentrations inhibited the migration of HepG_2_ cells in a concentration-dependent manner. LH at 40 μM resulted in an 86% inhibition of wound closure compared with control cultures (Figure 2B). Transwell cell invasion assay exhibited that non-toxic doses of LH inhibited red fluorescence-labeled HepG2 (RFP-HepG2) cells invasion in a concentration-dependent manner (Figure 2C and 2D). RFP-HepG2 treated with empty liposome can degrade matrigel and invade to the underside of filter. But LH at 40 μM could obviously inhibit the invasion of RFP-HepG2 cells. All these results suggested that non-toxic concentration of LH inhibits HepG2 cells motility and migration.

### Non-cytotoxic concentration of LH reduces the extravasation of HepG2 cells in zebrafish metastasis model

**Figure 3:**
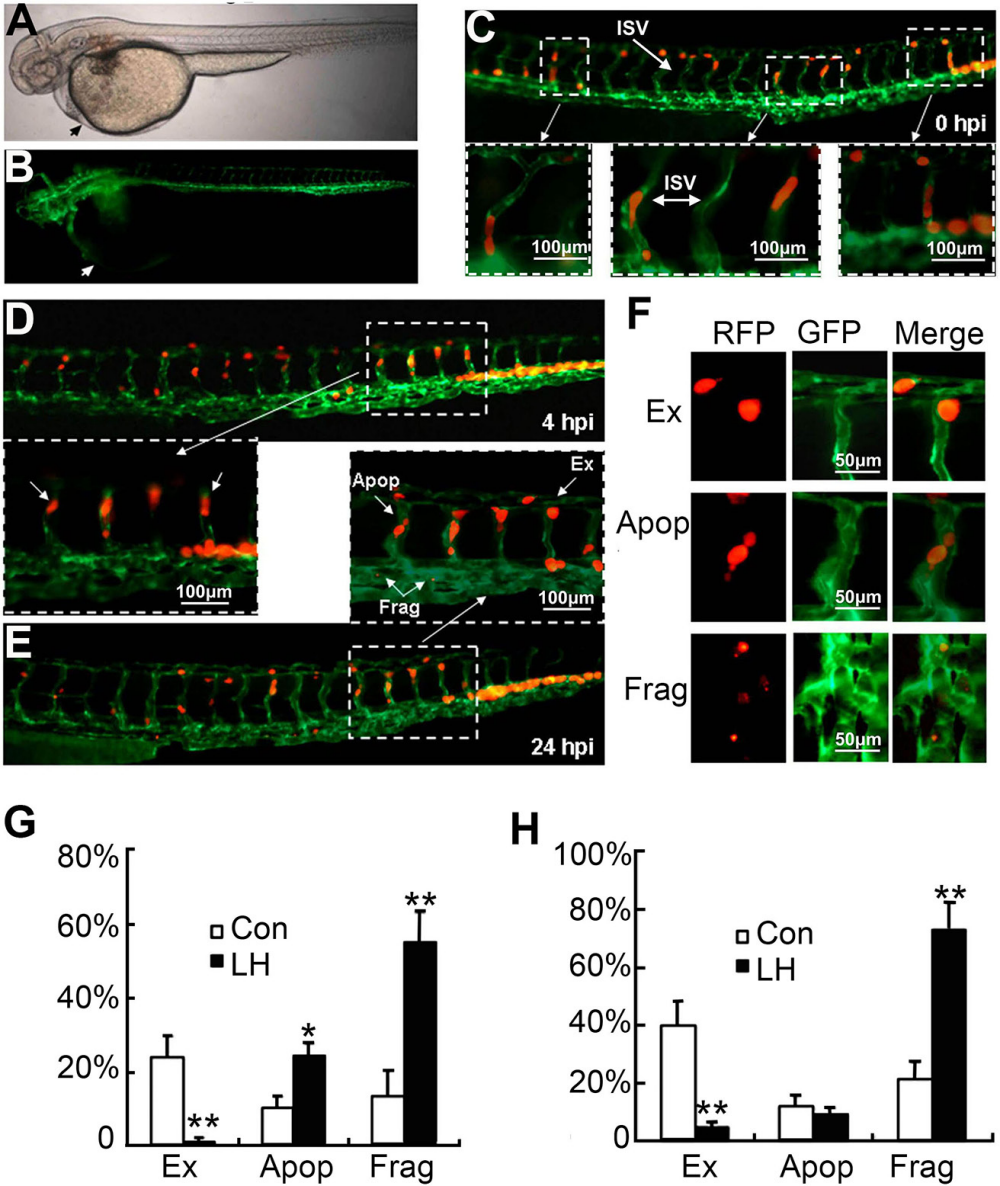
The effect of LH on extravasation of RFP-HepG2 cells in a metastasis model in Tg(flk1:EGFP) transgenic zebrafish (**A** and **B**) Tg(kdr1:EGFP) transgenic zebrafish at 3 days age, the arrows indicated the position where RFP-HepG2 cells were injected. A: white light; B: GFP labeled vascular system. (**C**) RFP -HepG2 cell entered the internode small vessels (ISV). RFP-HepG2 cells pretreated with empty liposome or LH (40 μM) for 6 hours were re-suspended in FCS-free DMEM media (2 × 10^7^/ml) and kept on ice for injection. RFP HepG2 cells were injected into the blood circulation (50–100 cells per fish) and then imaged at 0 h. Up: Image of RFP-HepG2 cells in ISV (200×). Below: Enlarge images showed RFP-HepG2 cells in ISV. (**D** and **E**) The extravasation of RFP-HepG2 cell in Tg (flk1:EGFP) zebrafish were observed and counted under a Zeiss fluorescent microscope (200×) at 4 h (D) and 24 h (E) after cell injection respectively. More than 20 fishes containing about 160 cells were analyzed for each group. D (up) showed the whole ISV image at 4 h, D (below) showed enlarged ISV image at 4 h. E (below) showed the whole ISV image at 24 h. E (UP) showed enlarged ISV image at 24 h. Apop: Apoptosis cells; EX: extravasation cells; Frag: fragmented cells. (**F**) The identification of extravasation, apoptosis and fragmented cells at the time point of 24 h. (**G** and **H**) Quantification of the average number of extravasation, apoptosis and fragmented cells in control and LH-pretreated cells injected zebrafish (*n* =20). Data represent the mean ± SE (*P < 0.05,**P < 0.01, compared with the control group).

Since migration and extravasation is one of the most important steps during tumor metastasis. To clearly study the effect of LH on extravasation of HepG2 cells at non-toxic concentration, we established a novel metastasis model in Tg (kdr1: EGFP) transgenic zebrafish by injecting RFP-HepG_2_ cells into blood circulation (Figure 3A and 3B). The arrows indicated the position where RFP-HepG_2_ cells were injected. This metastasis model in Tg(kdr1:EGFP) transgenic zebrafish could make us investigate tumor cells extravasation on a single cell level. As shown in Figure 3C, fluorescence-labeled HepG_2_ cells successfully entered the internode small vessels (ISV) of transgenic zebrafish. After 24 h entering the internode small vessels, these RFP-HepG_2_ cells presented different cell fates (Figure 3D and 3E), some tumor cells remained quiescence, some developed apoptosis (Figure 3F, Apop) or fragmented (Figure 3F, Frag), some tumor cells with strong transfer ability penetrated and extravastated out of host vascular vessels and entered into adjacent tissues within 24 h (Figure 3F, Ex). Quantification of the average number of extravasation, apoptosis and fragmented cells in pretreated and un-pretreated cells injected zebrafish (Figure 3G and 3H), about 25% and 40% HepG2 cells escaped out of host vascular vessels and entered into adjacent tissues within 24 h and 48 h in untreated group, respectively, whereas only about 2.5% and 5% LH-pretreated HepG_2_ cells completed the extravasation process within 24 and 48 hours. At the same time, about 17% HepG2 cells fragmented within 24 h in untreated group, by contrast, about 72% honokiol-pretreated HepG_2_ cells fragmented. We observed there was a slight increase in apoptosis cells between untreated and LH-pretreated groups in 24 h observation. As non-toxic dose of LH did not induce apoptosis, we speculated that the apoptosis increasing in 24 h observation was due to the motility and invasion inhibition by LH and the cells can not get used to the new environment within 24 h. Hence, some cells went apoptosis. Further 48 h observation demonstrated that honokiol-pretreated HepG_2_ cells did not cause obvious increases in apoptosis, which implied that 48 h after injection was a suitable time for observation on this model. This novel tumor cells extravasation model based on a single cell level clearly confirmed our hypothesis that LH significantly inhibits the extravasation and survival of HepG2 cells at a non-toxic concentration.

### LH suppresses the lamellipodia formation of HepG2 cells

**Figure 4:**
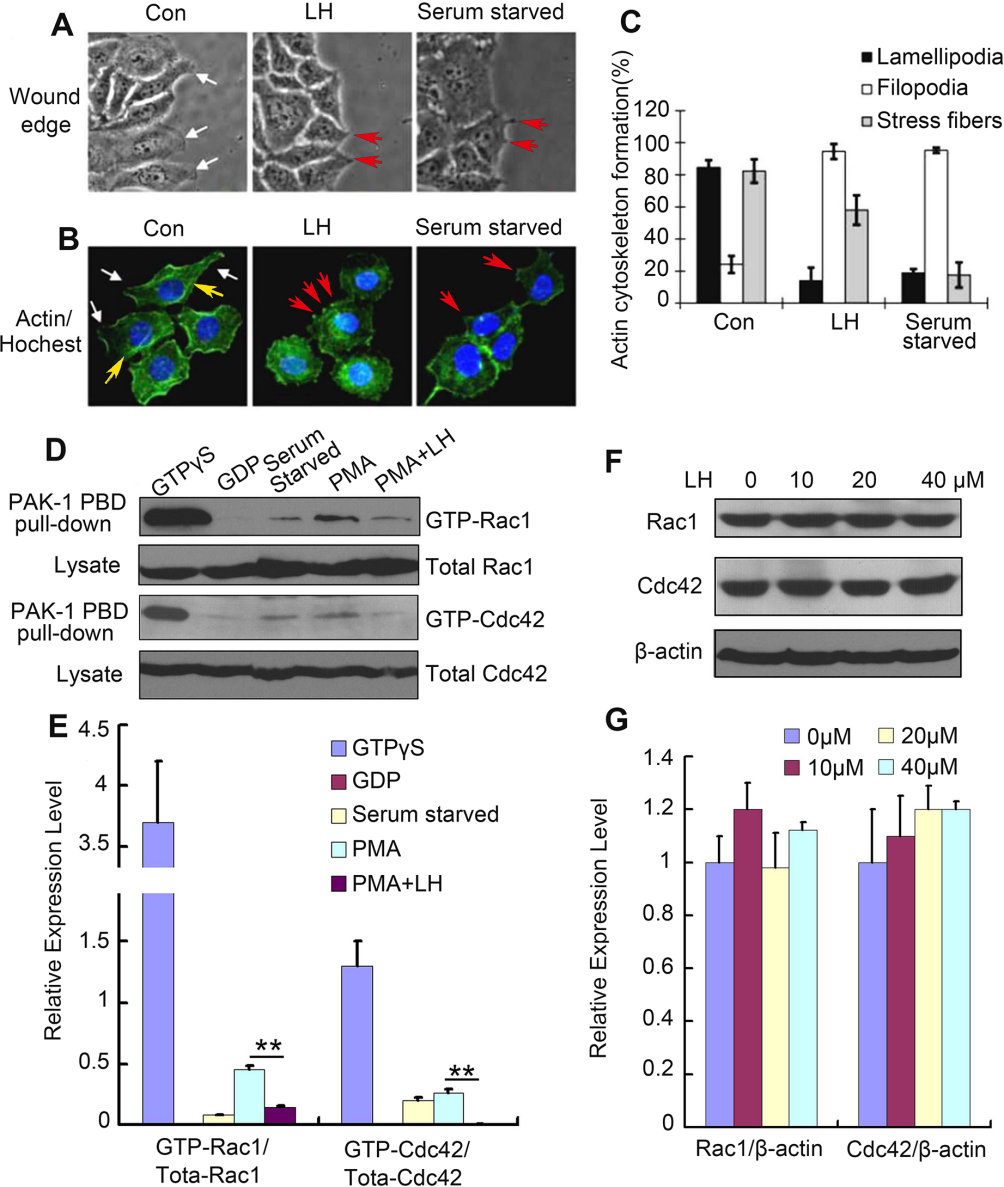
LH suppressed the lamellipodia formation and inhibited Rac1/Cdc42 activity in HepG2 cells (**A**) White light image HepG2 cells treated with empty liposome, 40 μM LH or serum starved for 24 h. lamellipodia were indicated by white arrows, filopodia were indicated by red arrows. (**B**)The immunofluorescence staining of FITC-conjugated phalloidin after 24 h treatment of empty liposome, 40 μM LH or serum starved in HepG2 cells. Then lamellipodia (white arrows), filopodia (red arrows) and stress fibers (yellow arrows) were counted respectively. The data are representative of 3 independent experiments. (**C**) Cumulative results of actin cytoskeleton formation from 3 independent experiments were shown as mean ± SE. (**D** and **E**). Immunoprecipitation of Rac1 and Cdc42. HepG2 cells were starved overnight before subjected to the indicated treatments, and then treated with GDP, PMA, and PMA+40 μM LH respectively. After 24 h treatment, the cells were lysed and immunoprecipitated with anti-GFP antibody, following by western blot assays of Rac1/Cdc42. The data are representative of 3 independent experiments and presented as the mean ± SE. **P < 0.01. (**F** and **G**) The effect of LH on the expression of Rac1 and Cdc42 in HepG2 cells using western blot analysis. The data are representative of 3 independent experiments and presented as the mean ± SE. Densitometric quantification of western blot was performed by Image QuantTL software.

Cell motility requires the tight temporal regulation of actin assembly and disassembly. Disregulated F-actin formation results in defects of cell motility abilities [33]. To test whether LH could inhibit HCC cells motility through affecting actin cytoskeleton formation, we observed the directly effect of LH on the morphology during migration and investigated the actin cytoskeleton of HepG2 cells using fluorescent phalloidin staining. HepG2 cells were starved in FCS-free DMEM medium overnight before cultured in 10% FBS DMEM with or without 40 μM honokiol. Under the stimulation of FCS, HepG2 cells extended broad lamellipodia into the open area in 6 hours (Figure 4A left and Figure 4B left), the lamellipodia in control cells were well organized. By contrast, LH-treated cell exhibited minimal lamellipodia and induced filopodia or microspikes formation of HepG2 cells (Figure 4A middle and Figure 4B middle), which was in accordance with the serum starved group (Figure 4A right and Figure 4B right). In 40 μM LH-treated group, cells with lamellipodia presented amount of 17% compared with 82% of control (Figure 4C, *p* < 0.01). LH at 40 μM also obviously increased the formation of filopodia and reduced the formation of stress fibers (Figure 4A–4C). Actin/Hoechst staining exhibited that the nuclear of honokiol-treated cells did not show obvious changes (Figure 4B), further confirming that the capability of LH in inhibiting the migration of HepG2 cells is correlated with the inhibition of the formation of lamellipodia but not induction of apoptosis.

### LH inhibits HepG2 motility via inhibiting Rac1/Cdc42 activity

It is well known that members of the Rho family of small GTPases, including RhoA, Rac 1, and Cdc42, are key regulators of the actin cytoskeleton in diverse cellular functions including cell migration [34]. To examine whether the effect of LH on HepG2 cells migration is mediated through inhibition of Rac 1 and Cdc42, we examined the activation of Rac1 and Cdc42 using GST pull-down assays in HepG2 cells. As shown in Figure 4D and 4E, control HepG_2_ cells showed high-levels of Rac1-GTP and Cdc42-GTP comparing to serum starved and GDP control cells. After stimulated with PMA, which activates Rac1 and thereby promotes cell spreading, lamellipodia formation, and cell migration, the levels of Rac1-GTP and Cdc42-GTP increased. However, after treatment with 40 μM LH, the increase of Rac1 and Cdc42 activities were significantly inhibited. Western blot showed that LH treatment had no obvious effect on the expression of Rac1 and Cdc42 (Figure 4F and 4G). These results further confirmed that inhibition of Rac1 and Cdc42 activity is the major cause of LH in the inhibition of HepG2 motility at non-toxic concentration.

### LH inhibits MMP-2/9 expression and enzyme activities

**Figure 5:**
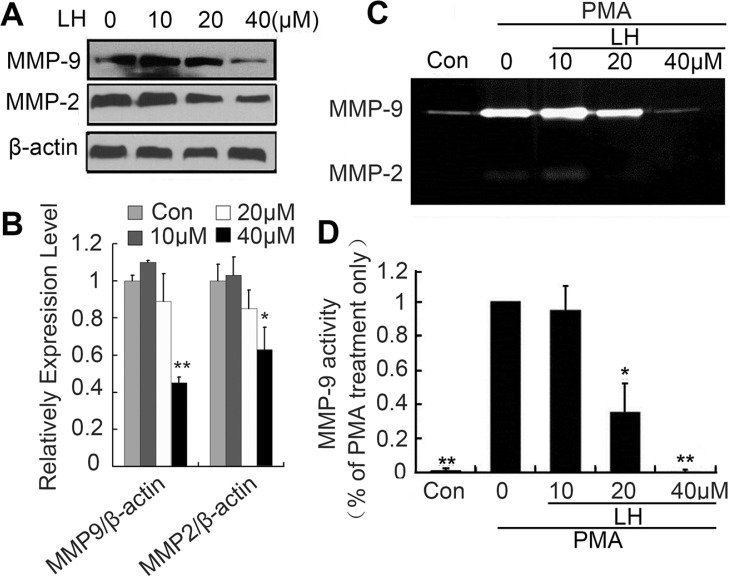
LH inhibited MMP-9 enzyme activity (**A** and **B**) HepG2 cells were treated with empty liposome or LH (10, 20, 40 μM) for 24 h, and then cell lysates were subjected to SDS-PAGE followed by western blots with anti-MMP-2, anti-MMP-9 antibodies. The data are representative of 3 independent experiments and presented as the mean ± SE, *P < 0.05, **P < 0.01, compared with the untreated group. (**C**, **D**) HepG2 cells were treated with empty liposome or LH (10, 20 and 40 μM) for 24 h in the presence of 200 nM PMA. MMP-9 and MMP-2 enzyme activities were analyzed by zymography (degradation the gelatin-containing polyacrylamide gel for 48 h). The data are representative of 3 independent experiments and presented as the mean ± SE, *P < 0.05, **P < 0.01, compared with the PMA treatment only. Densitometric quantification of gels was performed by ImageQuantTL software .

Matrix metalloproteinase-2 and -9 are two most important metastasis-promoting molecules, which involved several steps during the whole metastatic process at least including cell motility, invasion and extravasation [35, 36]. To explore the effects of LH on MMP-2 and MMP-9, we detected the activities and expression of MMP-2/9 using zymography and western-blot assay. The results revealed that LH not only down-regulated the expression of MMP-2/9 (Figure 5A and 5B), but also suppressed activities of PMA-induced MMP-2/9 at a concentration-dependent manner (Figure 5C and 5D). Non-toxic concentration at 40 μM LH completely inhibited the expression and activities of MMP-2/9.

### LH inhibits HepG2 cell motility by destabilizing EGFR and inhibiting the downstream PI3K/Akt, Erk, JNK pathways

**Figure 6:**
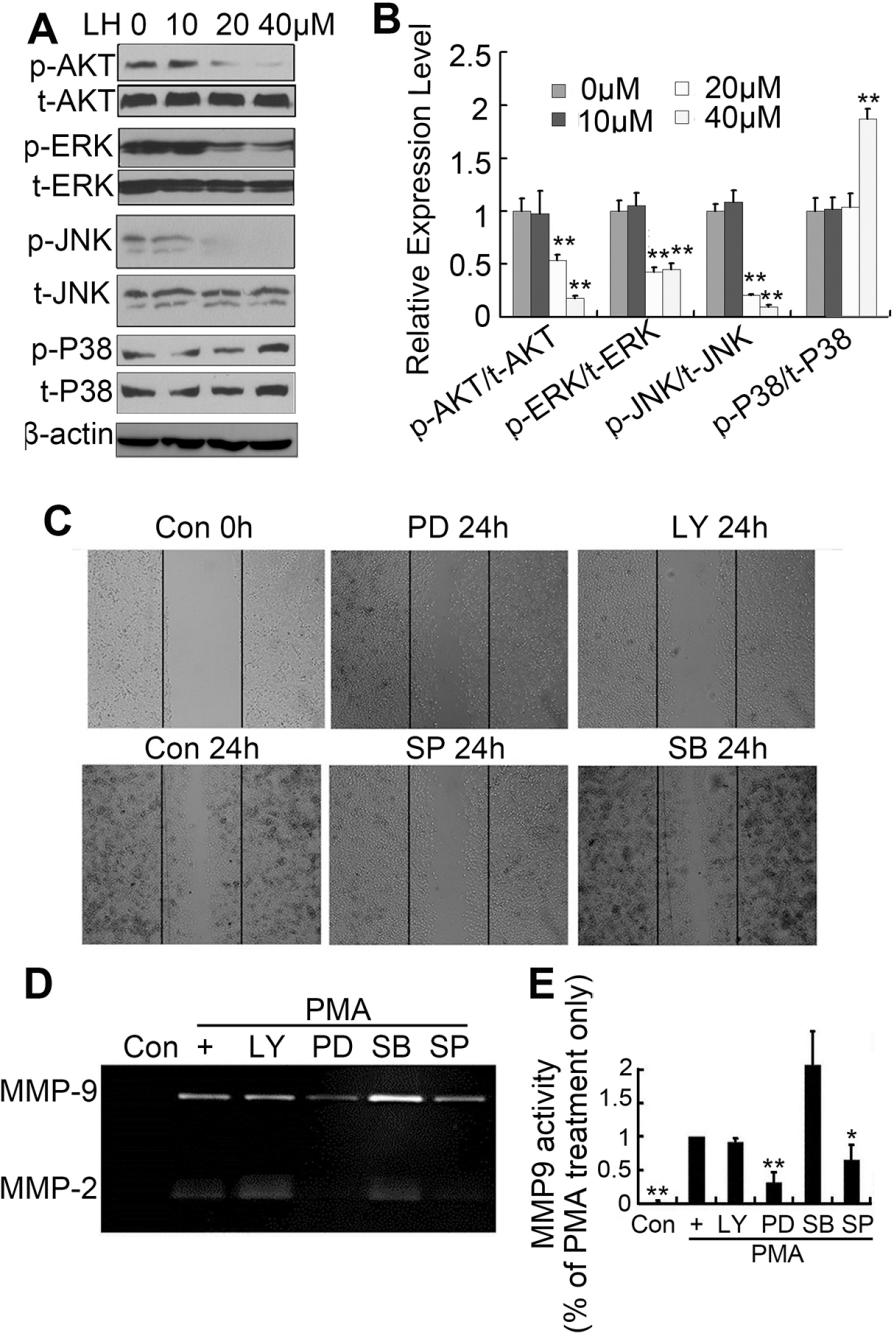
LH regulated the MAPK and PI3K/AKT pathways (**A** and **B**) HepG2 cells were treated with empty liposome or LH (10, 20, 40 μM) for 24 h, and then cell lysates were subjected to SDS-PAGE followed by western blots with anti-ERK, anti-P38, anti-JNK and anti-AKT (total and phosphorylation) antibodies. The data are representative of 3 independent experiments and presented as the mean ± SE, **P < 0.01, compared with the untreated group. (**C**) Cells were pretreated with 20 μM PD, LY, SB and SP followed by the addition of 200nM PMA for 24 h, and the migration abilities of cells were evaluated by wound-healing assays, the data are representative of 3 independent experiments. (**D** and **E**) Cells were treated with 20 μM PD, LY, SB and SP followed by the addition of 200 nM PMA for 24 h, the MMP-2 and MMP-9 levels were evaluated by gelatin zymography. the data are representative of 3 independent experiments and presented as the mean ± SE, *P < 0.05, **P < 0.01, compared with the PMA treatment only. Densitometric quantification of gels was performed by ImageQuantTL software.

In order to clarify the role of PI3K/Akt and MAPK pathways in cell motility, the activities of PI3K/Akt and MAPK pathways were studied by detecting phosphorylations of AKT, ERK, JNK and P38 using western blots. We found that LH obviously blocked the phosphorylations of AKT, ERK and JNK at a dose-dependent manner, but not P38 (Figure 6A and 6B). To explore the molecular mechanisms of LH to the cell migration and MMP enzymes in HepG2 cells, specific inhibitors of PI3K/Akt and MAPK pathways including PD98059 (an ERK inhibitor), SP600125 (a JNK inhibitor), SB203580 (a p38 MAPK inhibitor) and LY294002 (a PI3K inhibitor) were used in wound-healing assay and zymography assay. Wound-healing assay showed that the inhibition of PI3K pathway with LY294002 (20 μM) displayed the most potent inhibitory effects to the migration of HepG_2_ cells, SP600125 (20 μM) and PD98059 (20 μM) had little effect, whereas SB203580 (20 μM) had not effect (Figure 6C). Zymography assay showed that PD98059 (20 μM) significantly inhibited PMA-induced MMP-2/9 enzyme activities but not LY294002 and SP600125 (Figure 6D and 6E). In contrast, SB elevated PMA-induced MMP-2/9 enzyme activities. These data demonstrated that LH inhibited HepG2 cells migration predominately via PI3K/AKT pathway, and inhibited MMP enzymes activities mainly through ERK pathway.

**Figure 7:**
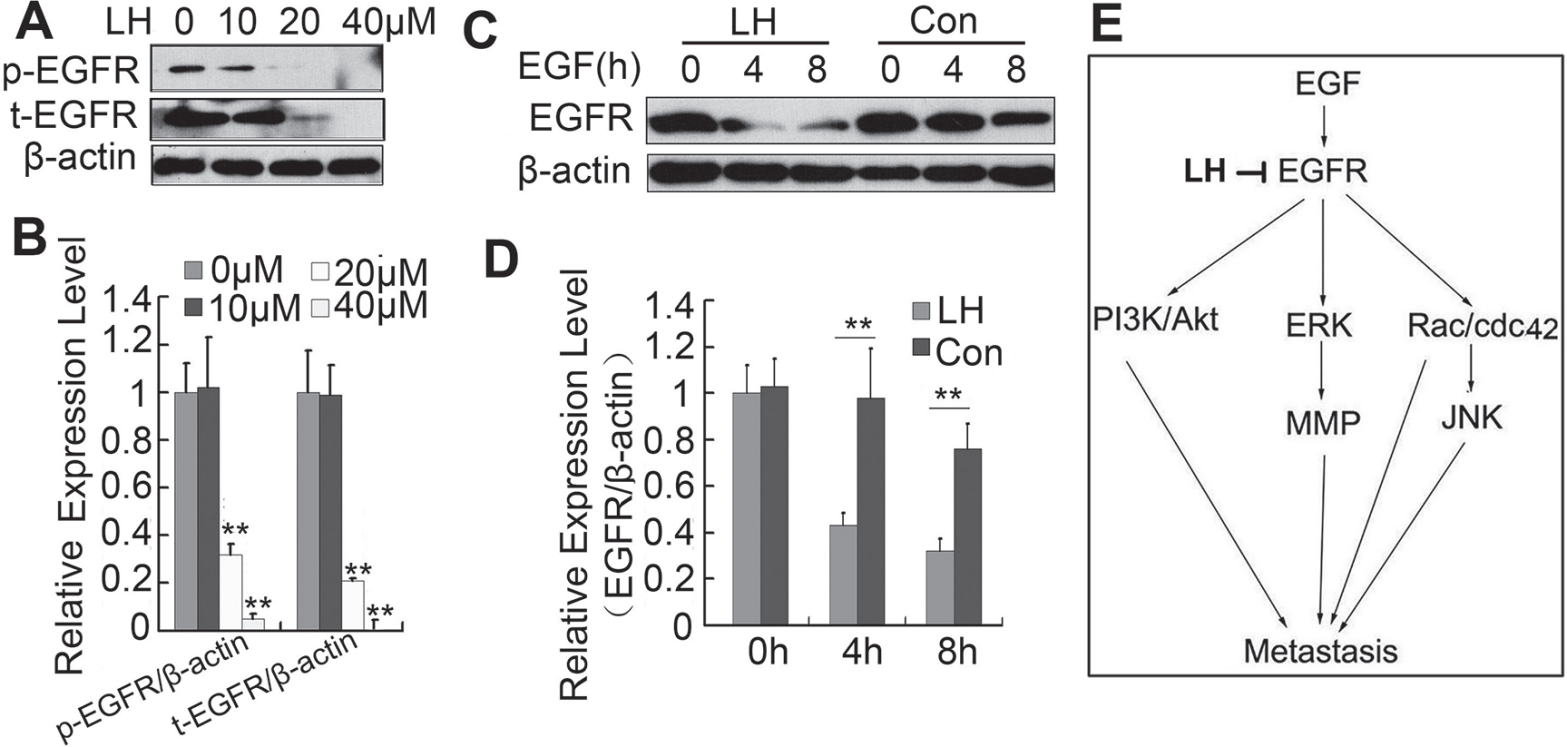
LH promotes the degradation of EGFR (**A** and **B**) HepG2 cells were treated with empty liposome or LH(10, 20, 40 μM) for 24 h, and then cell lysates were subjected to SDS-PAGE followed by western blots with anti-EGFR (total and phosphorylation). the data are representative of 3 independent experiments and presented as the mean ± SE, **P < 0.01, compared with the untreated group (**C** and **D**) HepG2 cells were deprived of growth factors for 12 hours and then exposed to EGF for the indicated time and the exposed to the indicated treatment, and then cell lysates were subjected to SDS-PAGE followed by western blots with anti-EGFR. The data are representative of 3 independent experiments and presented as the mean ± SE, **P < 0.01, compared with the untreated group. (**E**) Brief introduction of LH's inhibition on EGFR and downstream pathways. Densitometric quantification of gels was performed by Image QuantTL software.

In our attempt to investigate whether LH could affect EGFR phosphorylation level by western blot, we surprisingly found that LH inhibited the expression level of EGFR (Figure 7A and 7B), which implied that LH could inhibit the stability of EGFR. Further study showed that LH incubation accelerated EGFR degradation in the presence of EGF in HepG2 Cells (Figure 7C and 7D). This proved that LH could destabilize EGFR in HepG2 cells. As honokiol is reported to inhibit the expression of HSP90 [37] (a molecular chaperone that assists EGFR to fold and maintain conformation and stability), the degradation of EGFR reported by us may be the result of HSP90 inhibition by honokiol. With the above data together we could conclude that LH may inhibit HepG2 cell migration in a EGFR depend pathway: LH inhibits EGFR, promotes its degeradation, and inhibits the downstream PI3K/Akt, Erk, JNK pathways (Figure 7E), but the deeper mechanism still remains further study.

### LH inhibits hematogeneous metastasis of HepG2 and B16F10 cells in mice model

**Figure 8:**
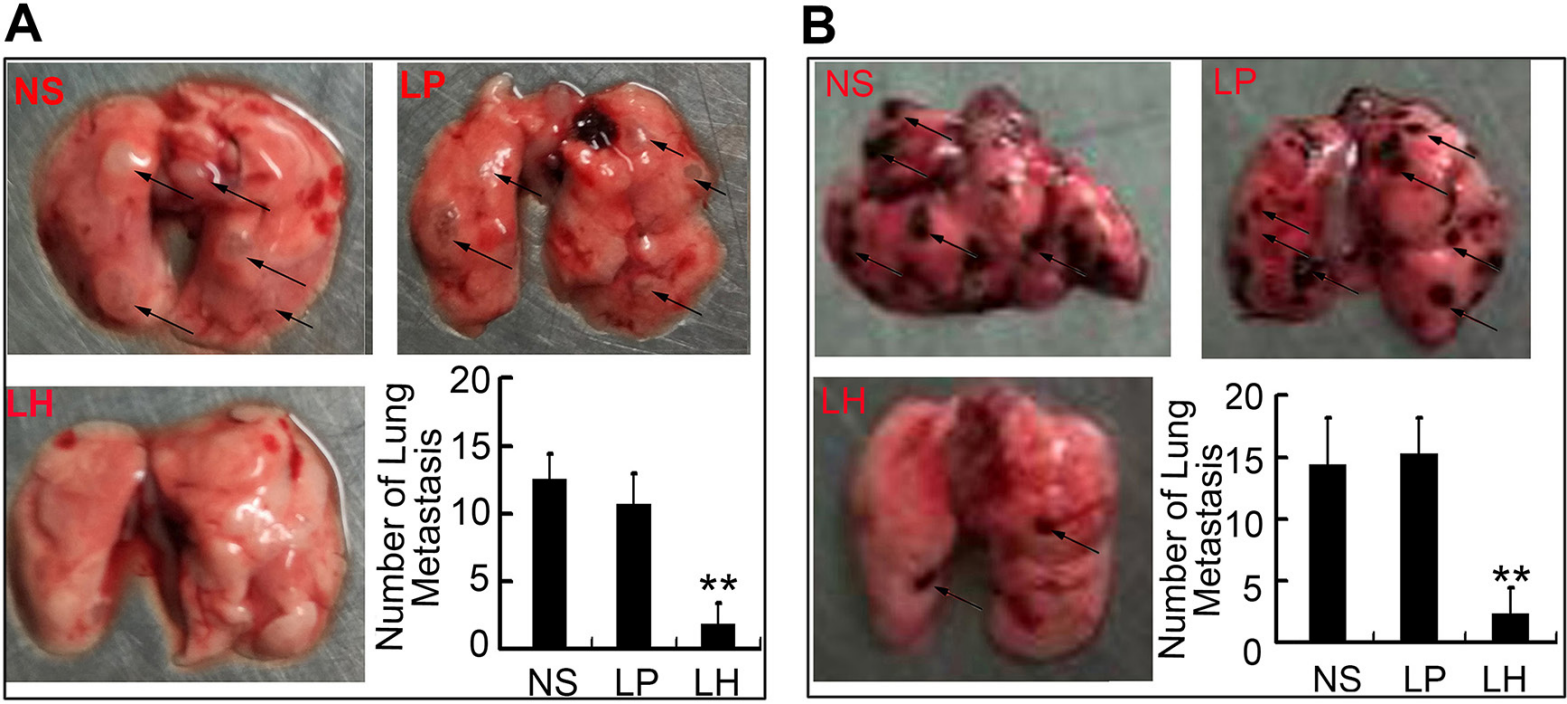
LH inhibits hematogeneous metastasis in HepG2 model (**A**) LH inhibited metastasis to lung by HepG2 cells in nude mice. 2 × 10^6^ HepG2 cells were i.v. injected into mice. Normal saline, empty liposome or LH (20 mg/kg) was given i.v. every 3 days for 15 d from the day of cells injection. After animals sacrificed, lungs metastasis nodes were imaged and counted. Metastasis nodes were indicated by dark arrows. Data represent the mean ± SE (**P < 0.01, compared with the NS group). (**B**) Mice were injected with B16F10 melanoma cells and left untreated for 8 days to allow tumor growth, then treated for another 15 days with normal saline, empty liposome or LH (20 mg/kg), respectively. After animals sacrificed, lungs metastasis nodes were imaged and counted, metastasis nodes were indicated by dark arrows. Data represent the mean ± SE (**P < 0.01, compared with the NS group).

In order to confirm the anti-metastasis potency of LH *in vivo* at low concentration, we established two lung metastasis models of HepG2 cells in nude mice and evaluated the therapeutic effects of LH. In the first lung metastasis model, nude mice were injected with HepG_2_ cells (2 × 10^6^/mouse) through the tail vein and treated with LH i.v injection. As shown in Figure 8A, mice treated with normal saline or empty liposome developed obvious pulmonary metastatisis, whereas LH treatment at 20 mg/kg i.v adminstration led to a significant inhibition of pulmonary metastasis.

Then we intend to investigate the effect of LH on B16F10 metastasic model *in vivo*. Mice were injected with B16F10 melanoma cells and left untreated for 8 days to allow tumor growth. Mice were then treated for another 15 days with normal saline, empty liposome or 20 mg/kg LH, respectively. The same results were obtained in B16F10 mice model. LH significantly inhibited the lung metastases (Figure 8B).

### LH inhibits pulmonary metastasis of HepG2 cells in mice model

**Figure 9:**
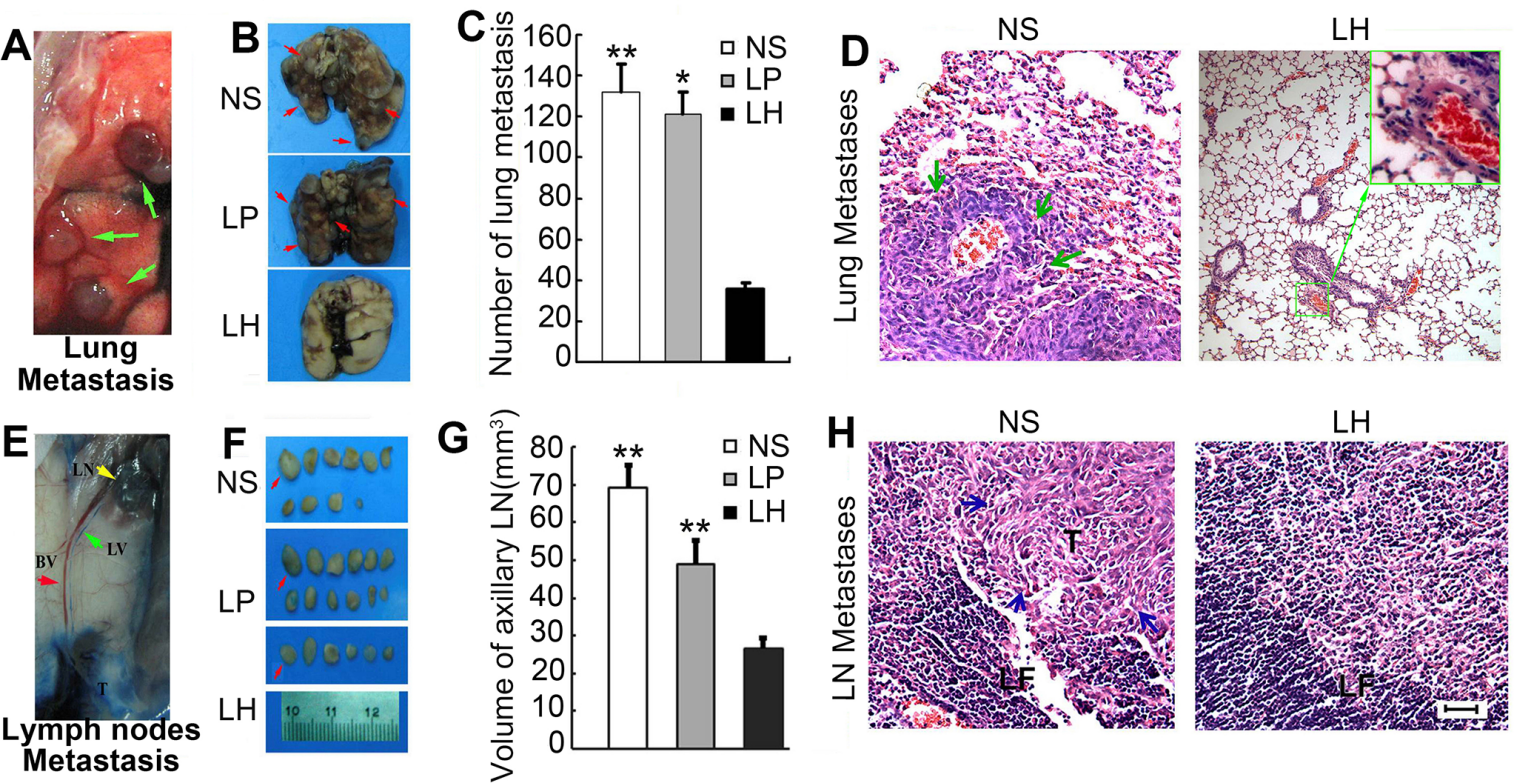
Suppression of pulmonary metastasis and regional lymph nodes metastasis by LH treatment in HepG2 bearing mice Animals (*n* = 5) were sacrificed on day 34 after cells injection. (**A**) Identification of lungs metastasis nodes (green arrow). (**B**) Three indicated groups of lungs metastasis nodes (arrows) were imaged. (**C**) Numbers of metastasis nodes on the lung surfaces. Data represent the mean ± SE (*P < 0.05, **P < 0.01, compared with the LH group). (**D**) The metastatic lesions in lungs were further confirmed by H&E staining, indicated by green arrows. (**E** and **F**) After Evans blue staining, the lymph nodes were sacrificed and imaged. E: Axillary lymph node with metastasis. Arrows (red: axillary lymph node, LN: lymph node, LV: lymphatic vessel, BV: blood vessel), T (tumor section). F: Regional lymph node images. (**G**) Calculation of total volumes of lymph nodes from each mouse. Data represent the mean ± SE. **P < 0.01, compared with the LH group. (**H**) Mice with regional lymph node metastasis confirmed by H&E staining. T indicates tumor section and pointed by blue arrows; TF: Folliculi lymphaticus.

We also established a spontaneous metastasis model by injecting HepG_2_ cells subcutaneously into the right flanks of female nude mice as description in Materials and Methods. As depicted in Figure 9A and 9B, all mice treated with either normal salines or empty liposome developed obvious metastatic lesions in lungs, whereas LH treatment at 20 mg/kg i.v adminstration led to a significant inhibition of pulmonary metastasis. The average lung surface metastasis nodes as counted in microscope, in NS group and empty liposome group were respective 34.4 metastases/mouse and 30.8 metastases/mouse. However, only 1 of the 5 mice on LH-treated group observed lung metastasis, and about 36 lung surface metastasis nodes were all counted in this mouse (Figure 9C). The metastatic lesions in lungs were further confirmed by H&E staining (Figure 9D), 4 of 5 mice in NS group and 4 of 5 mice in empty liposome group comparing to 1 of 5 mice in LH-treated group were definite obvious lung metastases.

### LH inhibits lymphatic metastasis of HepG2 cells in mice model

To further examine the capability of LH of inhibiting lymphatic metastasis, we examined lymph nodes metastasis of intradermal primary tumor by intradermal injecting evans blue (5 mg/ml in PBS), a dye that specifically stains lymphatic vessels and lymph nodes, by the side of tumor masses as previously described [26]. Then the lymph nodes of axillary, brachial, inguinal, popliteal and superficial cervical were stripped out. As Figure 9F shown, we observed a noticeable inhibition in the incidence of lymph node metastasis in LH-treated mice. The lymph nodes number of NS group and liposomal group were average 10.8 and 10 respectively, but the number of average lymph nodes in LH-treated group was only 6. A clear enlargement of the draining lymphatic vessels and lymph nodes were observed when Evans blue was injected intradermally by the side of tumor in the control group (Figure 9E). In contrast, there was no tumor-associated lymphatic vessel in 20 mg/kg LH-treated animals, which was correlated with a lack of dye-positive lymphatic vessels and lymph nodes. Furthermore, lymph node volumes of axillary were calculated. Figure 9G showed that the representative ipsilateral axillary lymph node volumes from HepG2-bearing mice. The mean lymph node volume was 27.9 ± 3.1 mm^3^ in LH-treated group (*n* = 5 lymph nodes total) compared with the mean volume of 69.1 ± 10.2 mm^3^ in NS group (*n* = 5, *P* < 0.01) and 49.1 ± 6.0 mm^3^ in empty liposome group (*n* = 5, *P* < 0.05). Also, the metastatic lesions in lymph nodes were further confirmed by H&E staining (Figure 9H). From the harvested lymph nodes, 4 of 5 mice in NS group, 3 of 5 mice in empty liposome group exhibited lymph node spread, whereas none of the 5 mice from LH-treated group exhibited lymph nodes metastases.

**Figure 10:**
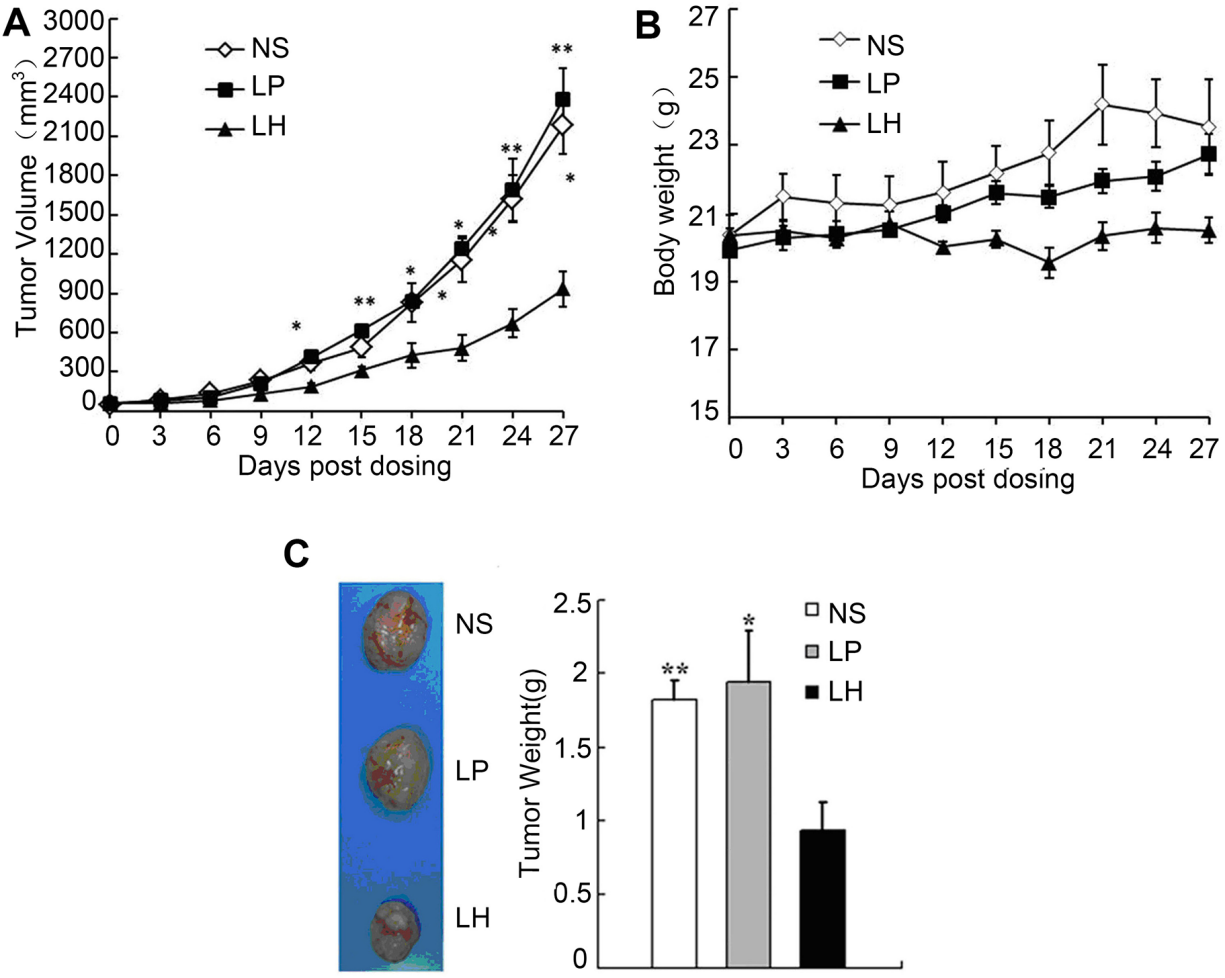
Antitumor effects of LH on HepG2 xenograft model Nude mice (*n* = 10 per group) were administered i.v. every 3 days with 20 mg/kg LH for 4 weeks starting at day 7 after 2 × 10^6^ HepG2 cells were injected into mice. (**A**) Indicate a significant difference in tumor volume between LH-treated groups and control groups. Points, average tumor volume; bars ± SE. Data represent the mean ± SE (*P < 0.05, **P < 0.01, compared with the LH group). (**B**) Toxicity-dependent weight loss in mice treated with LH compared with control groups. Points, average body weights; bars ± SE. (**C**) Tumor images of HepG2 xenograft models (left) and tumor weights of HepG2 xenograft models(right). Data represent the mean ± SE. *P < 0.05, **P < 0.01, compared with the LH group.

In addition, LH obviously inhibited tumor growth in HepG2 tumor-bearing nude mice model (Figure 10). After 24 days of LH treatment, the mean tumor volume was 734.437 ± 136.36 mm^3^ versus 1574.707 ± 194.07 mm^3^ in liposome-treated mice, and 1568.537 ± 230.49 mm^3^ in PBS-treated group, indicating a 50% inhibitory rate of tumor volume. These data indicated that LH significantly inhibited tumor growth and lung metastasis of HepG_2_ cells bearing mice model.

**Figure 11:**
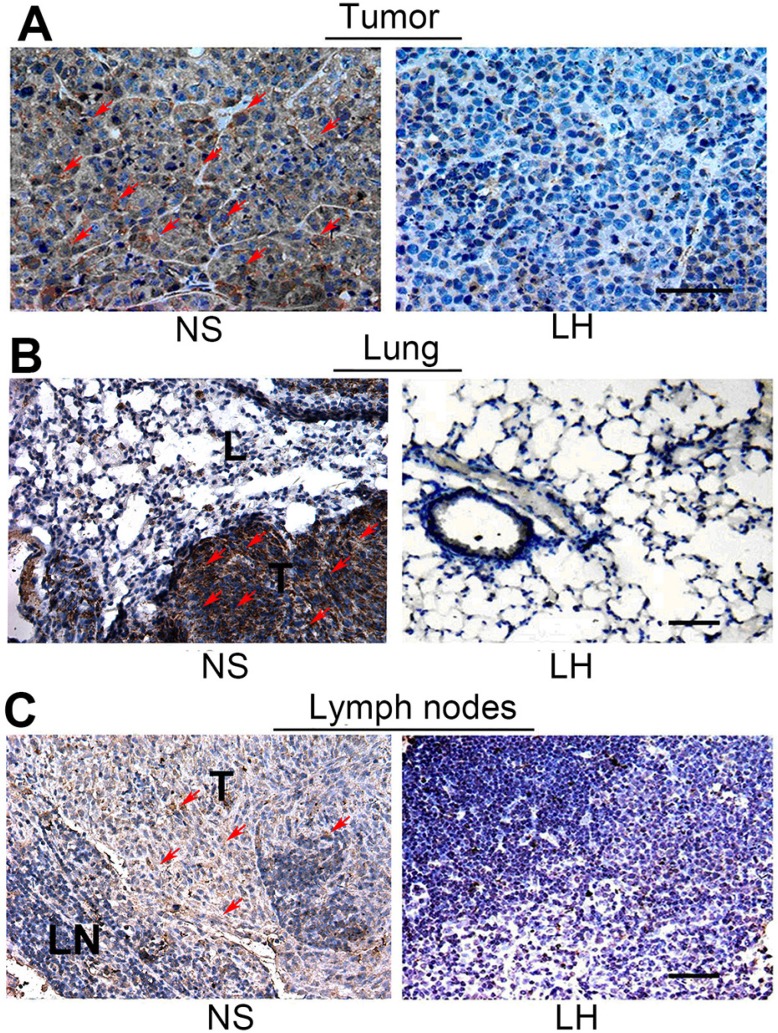
Assessment of the MMP-9 secretion in tissues of primary tumors, lungs and regional lymph nodes Paraffin-embedded sections were subjected to immunohistochemical analysis with MMP-9 antibody as described in materials and methods. (**A**) Expression in tumor sections. (**B**) Expression in lymph nodes sections. (**C**) Expression in lung sections. Partial staining-positive cells were indicated by red arrows.

To determine the effect of LH on the secretion of MMP-9 by HepG2 cells in primary tumor and metastatic lesions, tissue sections were assessed by immunohistochemistry of goat-anti-MMP-9 antibody (Figure 11). After the LH treatment, the expression of MMP-9 was obviously suppressed in primary tumor, while no positive expression of was found in the tissue of lymph nodes and lungs without metastatic tumor cells.

### Observation of potential toxicity

To evaluate the health status of mice treated with LH and empty liposome, weight of mice was monitored every 3 days throughout the whole experiment and considered a parameter for evaluation of weight loss, ruffling of fur, life span, behavior, or feeding and no significant difference in weights was found among the three groups (Figure 10B). In addition, no pathologic changes of liver, lung, kidney, spleen, brain, heart, or bone marrow were found by microscopic examination. In the long-term toxicity study of liposome honokiol on SD mouse and beagle dog (3.75, 7.5, 15 mg/kg/d on SD mouse and 4, 12, 40 mg/kg/d on beagle dog for four weeks), behaviors, blood chemistries and detailed histopathology of mice and dog were normal, no deaths occurred (data not shown).

## DISCUSSION

In the present study, we for the first time demonstrated that LH possessed potent anti-metastasis properties for HCC cells at non-toxic concentrations by destabilizing EGFR and inhibiting the downstream metastasis related PI3K/Akt, ERK and JNK pathway, which maybe a non-toxic anti-metastasis drug in a clinical setting.

We firstly identified that 40 μM LH is a non-toxic concentration on HCC cells. Although Rajendran *et al* reported that 25 μM honokiol treatment for 24 h could reduce HepG2 cell viability [31], our results still demonstrated that 40 μM LH is a non-toxic concentration on HCC cells. The different results may due to different generations of HepG 2 cells and culture medium. Honokiol was also reported to show toxic to many other cancer cells [17–27], but our results show LH (≤ 40 μM)showed no toxic to HCC cells, that was because honokiol may have many molecular targets in cancer cells and different cancer cells relays on different signal pathways for survival, so honokiol could show different activities in different cancers.

During the whole process of metastasis, including detachment from primary tumors, migration toward and entering into host vessels, and extravasating from circulation, the motility ability of tumor cells plays a fundamentally central role. Inhibiting cell motility is proposed as a promising strategy to prevent and suppress tumor metastasis [6]. We found non-toxic LH significantly inhibited the motility of HCC cell *in vitro*, and decreased the extravasation in zebrafish metastatic model. Cell migration is considered as a highly integrated multistep cycle process. The migratory cycle includes cell polarization, extension of protrusions in the direction of migration, formation of stable adhesion near the leading edge of the protrusions, and detachment of the adhesion and retraction at the rear. All these steps require tightly regulated changes of the actin cytoskeleton [38]. Rho family of small GTPases is a key regulator of the actin cytoskeleton in diverse cellular functions including cell migration. Rac1 is activated at the leading edge of motile cells and induces the formation of actin-rich lamellipodia protrusions, which serve as a major driving force of cell movement. Cdc42 were proved to regulate the organization of filopodia as the sensor of motile stimuli [33, 34, 38]. We found LH at non-toxic concentrations significantly affected actin cytoskeleton of HepG2 cells and interrupted the formation of filamentous F-actin stress fiber. The GST-Pak1-PBD pull down assay clearly showed that activities of Rac1 and CDC42 were inhibited by non-toxic LH. Taken together, our data indicated that the dysfunction of Rac1 and CDC42 might be the major cause of the interruption of actin cytoskeleton in non-toxic LH-treated HepG2 cells, and finally resulted in defects of motility ability and ineffectiveness of extravasation of HepG2 cells.

Interactions between tumor cells and extracellular matrix components (ECM) also existed in the whole metastatic process. Degradation and remodeling of extracellular matrix are necessary steps for tumor cells local migration, invasion and extravasation [35, 39, 40]. The major family of enzymes degrading extracellular matrix is matrix metalloproteinases (MMP) including MMP-9 and MMP-2. LH at non-toxic concentration displayed potent inhibitory ability to MMP-2 and MMP-9 expression and activities. Specific inhibitors of ERK and JNK also showed inhibitory effects to MMP-2 and MMP-9, suggesting ERK and JNK pathway might play an important role for LH activities. The results are consistent to previous studies about molecular explanation to honokiol activities. The signal pathway study showed that LH could destabilize EGFR and inhibit the down stream PI3K/AKT, ERK, JNK pathway, which resulted in the inhibition of Rac1/Cdc42, and MMP2/9 activity and finally cause the inhibition of metastasis with no obvious toxicity. So, our study also provide a robust evidence for that non-toxic EGFR or downstream pathway inhibitors will be effective anti-metastasis drugs in HCC. Besides, inhibition of EGFR signal pathway by non-toxic dose of LH results in HCC metastasis inhibition but causes no cell viability reduction, suggesting HCC cells needs EGFR for metastasis but their proliferation is not so sensitive to EGFR inhibition, implying HCC cells do not rely on EGFR for proliferation.

Given the pivotal functions of cytoskeleton remolding, cell motility and MMPs during tumors metastasis, it is easy to well understand the inhibitory activities of LH to HepG2 metastasis. It was further confirmed in nude mice model *in vivo*. The results showed LH remarkably inhibited pulmonary metastasis of HepG2 cells and resulted in an enhanced survival of the tumor bearing mice. Furthermore, we interestingly found LH also exhibited significant inhibition of regional lymph nodes metastasis. This also can be explained by the inhibition of actin cytoskeleton, cell motility and MMPs. The other reason of LH suppressing lymphatic metastasis might be the inhibition of VEGF-D-induced lymphangiogenesis via VEGFR-3 pathway [29, 41].

In conclusion, our study proved that non-toxic dose of LH can significantly suppress HCC metastasis with less toxicity to host body. Hence, LH may be a promising candidate agent for anti-metastasis in a clinical setting without causing severe side effects.

## MATERIALS AND METHODS

### Antibody and reagents

Anti-MMP-9 polyclonal goat antibody, anti-β-actin monoclonal mouse antibody, goat anti-rabbit IgG/HRP, goat anti-mouse IgG/HRP, rabbit anti-goat IgG/HRP were purchased from Santa Cruz Biotechnology (Santa Cruz, CA). Anti-Phospho-EGFR rabbit mAb, anti-EGFR rabbit mAb, anti-p44/42 MAP Kinase monoclonal rabbit antibody, anti-Phospho-p44/42 MAP Kinase monoclonal rabbit antibody, anti-AKT monoclonal rabbit antibody, anti-Phospho-AKT monoclonal mouse antibody, anti-Phospho-SAPK/JNK (Thr183/Tyr185) (81E11) Rabbit mAb, anti-SAPK/JNK rabbit mAb, anti-Phospho-p38 MAPK (Thr180/Tyr182) (12F8) Rabbit mAb and anti-p38 MAPK rabbit antibody were purchased from Cell Signaling Laboratories (Beverly, MA). PMA (Phorbol-12-myristate-13-acetate), specific inhibitors of PI3K [LY294002 (LY)], MAPK family [PD98059 (PD), mitogen-activated protein kinase kinase (MEK) inhibitor; SP600125 (SP), c-jun N-terminal kinase (JNK) inhibitor and SB203580 (SB), P38 MAPK inhibitor], gelatin and FITC-phalloidin were purchased from Sigma Chemical Co, (St.Louis, MO). PC, cholesterol and PEG4000 were purchased from Sigma Chemical Co, (St.Louis, MO); Honokiol was separated and purified by our laboratory and its purity and structure were analyzed and identified by high performance liquid chromatography and nuclear magnetic resonance [16].

### Cell lines and cell culture

Human hepatocellular carcinoma HepG2, SK-HEP1, SMMC-7721 cells, normal liver cells LO2 cells, mice melanoma B16F10 cells were obtained from the American Type Culture Collection (ATCC) in 2009. Cells were cultured in DMEM containing 10% fetal bovine serum (FBS), 100 U/mL penicillin, and 100 μg/mL streptomycin at 37°C in an atmosphere of 5% CO2. All the cells were tested and authenticated every year by an AmpFlSTR Identifiler PCR Amplification Kit (Applied Biosystems) in our laboratory, and the cells were last tested in October 2014. For visualizing dynamics of tumor cells metastasis process in zebrafish model, HepG2 cells were transfected with red fluorescent genes (pDsRed-N1 expression vectors; Invitrogen) by using Lipofectamine 2000 reagents. Then cells were selected via a limiting dilution protocol using 96-well plate. Briefly, transfected HepG2 cells were cultured in conditioned DMEM medium containing 700 μg/mL G418. After 7 days, G418-resistant cells were collected by trypsinization and amplified for use.

### Preparation of liposomal honokiol

To improve the poor water solubility of honokiol, liposomal honokiol (LH) was prepared in our laboratory and the method was described in our previously published paper [29]. The dose of LH was measured in equivalent dose of honokiol.

### Cytotoxicity assay

MTT assay was performed to evaluate the drug cytotoxicity. Cells were treated with empty liposome or various concentrations of LH in 96-well culture plates for 24 hours in final volumes of 200 μL (5 × 10^4^ cells/mL). Then 20 μL of MTT (5 mg/mL in PBS) was added to each well, incubated for an additional 4 h, the plate was centrifuged at 1000 rpm for 5 min, and then the medium was removed. MTT formazan precipitate was dissolved in 150 μL of DMSO, shaken mechanically for 5 min and then absorbance readings at a wavelength of 570 nm were taken on a spectrophotometer (Molecular Devices, Sunnyvale, USA).

### Cell cycle analysis

Cells were plated on 6-well culture plates. After incubation for 24 hours, cells were treated with empty liposome or 40 μM LH for another 24 hours. Cells were washed with PBS for two times, then fixed with 75% ethanol for 24 hours. Then cells were washed with PBS for three times, and stained with PI (50 μg/ml) for 20 min. The cells were then subjected to flow cytometry for cell cycle analysis.

### Annexin V-FITC/PI apoptosis assay

Cells were plated on 6-well culture plates. After incubation for 24 hours, cells were treated with empty liposome or 40 μM LH for another 24 hours. Cells were washed with PBS for two times. Then cells were subjected to the AnnexinV/PI Apoptosis Detection kit (Invitrogen) for staining according to the manufacturer's instructions, and finally analyzed by flow cytometry.

### Cell migration analysis

Wound-healing assay was carried out to examine the migration potency of tumor cells. Briefly, HepG2 cells were seeded in gelatin-coated 6-well plates and grown overnight to confluence. The monolayer cells were scratched with 200 μL pipette tips to create wounds, and cells were washed twice with serum-free DMEM to remove the non-adherent cells and then replaced with serum-free DMEM. Cells were subjected to the indicated treatment for 24 h, and cells migrating from the leading edge were photographed immediately after wounding (0 hour) and at time points 24 hours. Average rates of wound closure were derived from three randomly selected fields.

### Immunofluorescence staining

HepG2 HCC cells were allowed to grow on glass coverslips in Dulbecco's modified Eagle's medium supplemented with 10% calf serum (complete growth medium). Serum was starved overnight, and then subjected to the indicated medium for 24 h before they were treated with empty liposome or LH (40 μM). Cells on the coverslips were washed with ice-cold PBS and fixed with 4% paraformaldehyde solution in PBS for 10 minutes. After washed with PBS 3 times, cells were permeabilized with 0.1% Triton X-100 in PBS for 5 minutes. After additional 3 times PBS washing, cells were incubated with FITC-conjugated phalloidin in PBS (5 μg/ml) for 30 minutes, stained of nuclear by Hoechst 33258 (10 μg/ml) for 10 minutes and washed with PBS for 30 minutes. Coverslips were mounted in 90% glycerol containing 0.1%–0.1% p-phenylenediamine dihydrochloride in PBS. Images were captured using a Zeiss microscope equipped with a digital camera.

### Measurement of Rac1/Cdc42 activity by pull down assay

In brief, HepG2 cells were starved in serum free DMEM medium overnight before subjected to the indicated treatments. PMA were used as an activator of Rho family small GTP proteins. After ice-cold rinse and lyses, equal amount of lysate were incubated with GST-Pak1-PBD agarose in resins at 4°C for 1 hour. During this process, GTPγS (100 μM) and GDP (1 mM) protein were added to untreated lysates as positive and negative control respectively. The mixtures were centrifuged, washed and eluted in lyses buffer to pull down only the active form of Rac1 and Cdc42. The eluted samples were re-suspended in 2× Laemmli reducing sample buffer and boil for 5 minutes and run on 12% SDS-PAGE and subjected to Western blot assay using antibodies against Rac1 or Cdc42. Rac1 and Cdc42 activities were indicated by amounts of Pak1-PBD-bound Rac 1(GTP-Rac1) and Cdc42 (GTP-Cdc42). Total cell lysates were also subjected to immunoblotting for the levels of total Rac1 and Cdc42.

### Western blotting

This experiment was performed following previously study [42]. Cells were lysed with protein lysis buffer [150 mM NaCl, 1.0% Nonidet P-40, 0.5% sodium deoxycholate, 0.1% SDS, 50 mM Tris-HCl (pH 8.0), 5mM sodium fluoride, 1 mM sodium orthovanadate and 1mM phenylmethylsulfonyl fluoride (PMSF)]. Protein concentration was determined by the bradford protein assay. The samples were denatured in sample buffer, and equal amounts of protein were separated according to molecular weight on a 8% to 12% SDS-PAGE gel and transferred onto a polyvinylidene difluoride (PVDF) membrane. Membranes were blocked for 1 hour in 5% dried milk in TBST at room temperature and probed overnight at 4°C with indicated primary antibodies diluted in “blocking buffer”. Blots were washed thrice for about 15 minutes with TBST and incubated with a horseradish peroxidase–conjugated species-specific antibody diluted in blocking buffer for 1 hour at room temperature with rotation. After three additional washes, blots were developed by a 1-minute incubation with enhanced chemiluminescent substrate and exposure to Kodak X-OMAT autoradiographic film (Kodak, Hemel Hempstead, United Kingdom).

### Quantification of HepG2 extravasation in zebrafish metastasis model

To study the effect of honokiol on extravasation of HepG2 cells, we established a metastasis model in Tg(flk1:EGFP) transgenic zebrafish as previously described [43]. Briefly, 48 hours old Tg(flk1:EGFP) transgenic zebrafish embryos were obtained and maintained at 28.5°C. Embryos were de-chorionized (if necessary) and anesthetized with 0.003% tricaine and positioned on their right side on a wet agarose plate. Red fluorescence-labeled HepG2 cells (HepG2-Red) pretreated with empty liposome or LH (40 μM) for 6 hours were resuspended in FCS-free DMEM media (2 × 10^7^/ml) and kept on ice for injection. Pretreatment with LH was adapted instead of treatment after cells injection to avoid the potential interruption of zebrafish vascular system from LH. Then HepG2-Red cells were injected into the blood circulation (50–100 cells per fish) using a micro-injector equipped with a 0.75 mm glass needles (no filament, L = 50 mm, diameter of the needle opening = 20 μM). Zebrfish burdened tumor cells were maintained at 30.5°C. 24 and 48 hours after injection, the extravasation of HepG2-Red in Tg (flk1:EGFP) zebrafish embryos were observed and counted under a Zeiss fluorescent microscope (200×). More than 20 fish containing about 160 cells were analyzed for each group.

### Establishment of pulmonary metastasis models

Two pulmonary metastatic models were established in female athymic BALB/c nude mice (6–8 weeks of age) and C57BL6 mice (6–8 weeks of age) for the investigation of anti-metastatice effect of LH, according to the guidelines set by the Institute's Animal Care and Use Committee. During the experimental period, all animals were maintained in a dedicated aseptic environment as approved by institutional protocol and guidelines. In the first series of experiments, therapeutic effects on the early phases of metastasis were evaluated. HepG_2_ cells (2 × 10^6^) in 100 μL serum-free medium were injected through the tail vein of nude mice. The treatment initiated from the day of tumor cells injection. The mice were randomized into 3 groups (*n* = 6) and treated with 20 mg/kg LH, empty liposomal or normal saline, respectively. Each mouse was administered i.v. every three days for 15 days. After mice were sacrificed, lungs were collected for identify the number of metastasis nodules on the lung surface. Lung were excised and fixed in 10% formalin for further histologic analysis.

To assess therapeutic effects in already established metastases, C57BL6 mice (*n* = 6 mice per group) were injected intravenously with 2.5 × 10^5^ B16F10 cells, and treatment was started 8 days after transplantation. The mice were randomized into 3 groups (*n* = 6) and treated with 20 mg/kg LH, empty liposomal or normal saline, respectively. Each mouse was administered i.v. every three days for 15 days. Metastasis was evaluated 15 days after initiation of treatment.

### Establishment of lung metastasis and regional nodal metastasis tumor xenograft

To determine the *in vivo* anti-tumor activity of LH on lung metastasis and regional nodal metastasis, HepG2 cells (2 × 10^6^ in 100 μL saline) were injected s.c. into the right flanks of female nude mice (6 weeks old, BALB/cA-nu (*nu/nu*). When the tumor reached 3 mm × 3 mm, thirty mice were randomly divided into 3 groups (*n* = 10) and treated with 20 mg/kg LH, empty liposome and normal saline (NS), respectively. The treatment initiated at day 7 after tumor cells injection. Each mouse was administered i.v. every three days for 4 weeks. Tumor volume were measured every three days with a caliper (calculated volume (mm^3^) = π/6 × length × width × width). As a general measure of toxicity, body weights were determined on the same schedule as tumor volume measurements.

### Identification of pulmonary metastases and metastatic regional lymph nodes

Mice (*n* = 5) were sacrificed to identify the extrahepatic metastasis to the regional lymph nodes and lungs on day 34 after cells injection in the xenograft model. The rest of the mice (*n* = 5) were examined daily for survival until 60th day or sacrificed and regarded as dead when the tumor volume reached 4000 mm^3^. The presence of metastasis was macroscopically examined in all abdominal and thoracic internal organs in both two mice models. Lungs were removed and photographed, and then the number of surface metastatic nodules on lung specimens was counted under a micro-dissecting microscope. All specimens including regional lymph nodes and internal organs were evaluated by H&E staining to confirm the metastatic lesions.

To searching the regional lymph nodes, anesthetized HepG2 tumor-bearing mice were intradermally injected with 100 μL Evans blue (Sigma, 5 mg/ml in PBS) by the side of tumor. After 10 minutes, mice were photographed for observation of the dye going through the draining lymphatic vessels and Evans blue-stained draining lymph nodes. Following the lymphatic mapping, the axillary, brachial, inguinal, popliteal and superficial cervical lymph nodes were extirpated.

### Histologic analysis

Specimens of primary tumor with adjacent tissues, relevant internal organs and lymph nodes were harvested and fixed in 4% paraformaldehyde (PFA) for paraffin sectioning of H&E. And followed by staining in hematoxylin solution 5 minutes; washing in tap water; rinsed in HCl solution 0.1% for 10 s; washed in tap water; stained in eosin solution for 2 minutes; washed in tap water, then mounted sections in neutral gum.

### Statistical analysis

Data were assayed by ANOVA and unpaired student's *t-test*. Values are expressed as the mean ± SE. Survival curves were constructed according to the Kaplan-Meier method, and the survivals were compared by means of the log rank test. A *P-value* of < 0.01 or < 0.05 was regarded as indicating a significant difference.

### Ethics statement

All the methods were carried out in accordance with the approved guidelines and the entire experimental protocols were approved by the Institutional Animal Care and Use Committee of Sichuan University (Chengdu, China).

## References

[1] Hussain SA, Ferry DR, El-Gazzaz G, Mirza DF, James ND, McMaster P, Kerr DJ (2001). Hepatocellular carcinoma. Ann Oncol.

[2] Kensler TW, Qian GS, Chen JG, GroopmaTPAn JD (2003). Translational strategies for cancer prevention in liver. Nat Rev Cancer.

[3] Trevisani F, Cantarini M, Wands JM (2008). Recent advances in the natural history of hepatocellular carcinoma. Carcinogenesis.

[4] Katyal S, Oliver JH, Peterson MS, Ferris JV, Carr BS, Baron RL (2000). Extrahepatic Metastases of Hepatocellular Carcinoma. Radiology.

[5] Qin LX, Tang ZY (2002). The prognostic molecular markers in hepatocellular carcinoma. World J Gastroenterol.

[6] Chambers AF, Groom AC, MacDonald IC (2002). Dissemination and growth of cancer cells in metastatic sites. Nat Rev Cancer.

[7] Weber GF (2013). Why does cancer therapy lack effective anti-metastasis drugs?. Cancer Lett.

[8] Ji EL, Si HB, Choi JY, Yoon SK, You YK, Lee MA (2014). Epirubicin, Cisplatin, 5-FU combination chemotherapy in sorafenib refractory metastatic hepatocellular carcinoma. World J Gastroenterol.

[9] Wang Q, Zhong YJ, Yuan JP, Shao LH, Zhang J, Tang L, Liu SP, Hong YP, Raymond AF, Li Y (2013). Targeting therapy of hepatocellular carcinoma with doxorubicin prodrug PDOX increases anti-metastatic effect and reduces toxicity: a preclinical study. J Trans Med.

[10] Huang P, Xu X, Wang L, Zhu B, Wang X, Xia J (2014). The role of EGF-EGFR signalling pathway in hepatocellular carcinoma inflammatory microenvironment. J Cell Mol Med.

[11] Steeg PS (2003). Metastasis suppressors alter the signal transduction of cancer cells. Nat Rev Cancer.

[12] Teng CM, Chen CC, Ko FN, Lee LG, Huang TF, Chen YP, Hsu HY (1988). Two antiplatelet agents from Magnolia officinalis. Thromb Res.

[13] Clark AM, El-Feraly FS, Li WS (1981). Antimicrobial activity of phenolic constituents of Magnolia grandiflora L. J Pharm Sci.

[14] Watanabe K, Watanabe H, Goto Y, Yamaguchi M, Yamamoto N, Hagino K (1983). Pharmacological properties of magnolol and honokiol extracted from Magnolia officinalis: central depressant effects. Planta Med.

[15] Liou KT, Shen YC, Chen CF, Tsao CM, Tsai SK (1983). Honokiol protects rat brain from focal cerebral ischemia-reperfusion injury by inhibiting neutrophil infiltration and reactive oxygen species production. Brain Res.

[16] Chen L, Zhang Q, Yang G, Fan L, Tang J, Garrard I, Svetlana I, Derek F, Sutherland I (2007). Rapid purification and scale-up of honokiol and magnolol using high-capacity high-speed counter-current chromatography. J Chromatogr A.

[17] Wang T, Chen F, Chen Z, Wu YF, Xu XL, Zheng S, Hu X (2004). Honokiol induces apoptosis through p53-independent pathway in human colorectal cell line RKO. World J Gastroenterol.

[18] Hibasami H, Achiwa Y, Katsuzaki H, Imai K, Yoshioka K, Nakanishi K, Ishii Y, Hasegawa M, Komiya T (1998). Honokiol induces apoptosis in human lymphoid leukemia Molt 4B cells. Int J Mol Med.

[19] Konoshima T, Kozuka M, Tokuda H, Nishino H, Iwashima A, Haruna M, Kazuo I, Masahiro T (1991). Studies on inhibitors of skin tumor promotion, IX: neolignans from Magnolia officinalis. J Nat Prod.

[20] Ishitsuka K, Hideshima T, Hamasaki M, Raje N, Kumar S, Hideshima H, Norihiko S, Hiroshi Y, Aldo MR, Paul R, Klaus P, Steven LG, Dharminder C (2005). Honokiol overcomes conventional drug resistance in human multiple myeloma by induction of caspase-dependent and -independent apoptosis. Blood.

[21] Yang SE, Hsieh MT, Tsai TH, Hsu SL (2002). Down-modulation of Bcl-xL, release of cytochrome c and sequential activation of caspases during honokiol-induced apoptosis in human squamous lung cancer CH27 cells. Biochem Pharmacol.

[22] Hahm E, Singh SV (2007). Honokiol causes G0-G1 phase cell cycle arrest in human prostate cancer cells in association with suppression of retinoblastoma protein level/phosphorylation and inhibition of E2F1 transcriptional activity. Mol Cancer Ther.

[23] Hahm ER, Arlotti JA, Marynowski SW, Singh SV (2008). Honokiol, a constituent of oriental medicinal herb magnolia officinalis, inhibits growth of PC-3 xenografts *in vivo* in association with apoptosis induction. Clin Cancer Res.

[24] Xianhe B, Francesca C, Masuko UF, Muhammad W, Campbell PM, Baskaran G, Channing JD, Traci B, David AF, Ye KQ, Emma M, Wolfgang D, Gerald S (2003). Honokiol, a small molecular weight natural product, inhibits angiogenesis *in vitro* and tumor growth *in vivo*. J Biol Chem.

[25] Nagase H, Ikeda K, Sakai Y (2001). Inhibitory effect of magnolol and honokiol from Magnolia obovata on human fibrosarcoma HT-1080 invasiveness *in vitro*. Planta Med.

[26] Kwang SA, Gautam S, Shishir S, Bokyung S, Jack LA, Bharat BA (2006). Honokiol potentiates apoptosis, suppresses osteoclastogenesis, and inhibits invasion through modulation of nuclear factor-κB activation pathway. Mol Cancer Res.

[27] Liu Y, Chen L, He X, Fan L, Yang G, Chen X, Lin X, Du L, Li Z, Ye H, Mao Y, Zhao X, Wei Y (2008). Enhancement of therapeutic effectiveness by combining pegylated liposomal of honokiol and cisplatin in ovarian carcinoma. Int J Gynecol Cancer.

[28] Hou WL, Chen LJ, Yang GL, Zhou H, Jiang QQ, Zhong ZH, Hu J, Chen X, Wang XH, Tang MH, Wen J, Wei YQ (2008). Synergistic antitumor effects of liposomal honokiol combined with adriamycin in breastcancer models. Phytother Res.

[29] Wen J, Fu A, Chen L, Xie X, Yang G, Chen X, Chen XC, Wang YS, Li J, Chen P, Tang MH, Shao XM, Lu Y (2009). Liposomal honokiol inhibits VEGF-D-induced lymphangiogenesis and metastasis in xenograft tumor model. Int J Cancer.

[30] Hu J, Chen LJ, Liu L, Chen X, Chen PL, Yang GL, Hou WL, Tang MH, Zhang F, Wang XH, Zhao X, Wei YQ (2008). Liposomal honokiol, a potent anti-angiogenesis agent, in combination with radiotherapy produces a synergistic antitumor efficacy without increasing toxicity. Expl Mol Med.

[31] Rajendran P, Li F, Shanmugam MK, Vali S, Abbasi T (2012). Honokiol inhibits signal transducer and activator of transcription-3 signaling, proliferation, and survival of hepatocellular carcinoma cells via the protein tyrosine phosphatase SHP-1. J Cell Physiol.

[32] Min SS, Kim JH, Kim HJ, Chang KC, Sang WP (2015). Honokiol activates the LKB1–AMPK signaling pathway and attenuates the lipid accumulation in hepatocytes. Toxical Appl Pharm.

[33] Maruthamuthu V, Aratyn-Schaus Y, Gardel ML (2010). Conserved F-actin dynamics and force transmission at cell adhesions. Curr Opin Cell Biol.

[34] Parri M, Chiarugi P (2010). Rac and Rho GTPases in cancer cell motility control. Cell Commun Signal.

[35] Seiki M (2002). The cell surface: the stage for matrix metalloproteinase regulation of migration. Curr Opin Cell Biol.

[36] Liotta LA, Kohn EC (2001). The microenvironment of the tumour-host interface. Nature.

[37] Park EJ, Min HY, Chung HJ, Hong JY, Kang YJ, Hung TM, Ui JY, Yeong SK, Ki HB, Sam SK, Sang KL (2009). Down-regulation of c-Src/EGFR-mediated signaling activation is involved in the honokiol-induced cell cycle arrest and apoptosis in MDA-MB-231 human breast cancer cells. Cancer Lett.

[38] Jiménez C, Portela RA, Mellado M, Rodríguez-Frade JM, Collard J, Serrano A, Carlos MA, Jesus A, Ana CC (2000). Role of the PI3K regulatory subunit in the control of actin organization and cell migration. J Cell Biol.

[39] Robert RL, Isaiah JF (2007). Tumor Cell-Organ Microenvironment Interactions in the Pathogenesis of Cancer Metastasis. Endocr Rev.

[40] Johanna AJ (2005). Therapeutic targeting of the tumor microenvironment. Cancer Cell.

[41] Zhao CJ (2011). Distinct contributions of angiogenesis and vascular co-option during the initiation of primary microtumors and micrometastases. Carcinogenesis.

[42] Shen W, Du R, Li J, Shen WZ, Du RL, Li J, Luo XH, Zhao ST, Chang AT, Zhou W, Gao RF, L uo DH, Wang J (2016). TIFA suppresses hepatocellular carcinoma progression via MALT1-dependent and-independent signaling pathways. Signal Transd Target Ther.

[43] Stacker SA, Caesar C, Baldwin ME, Thornton GE, Williams RA, Prevo R, David GJ, Nishikawa S, Hajime K, Marc GA (2001). VEGF-D promotes the metastatic spread of tumor cells via the lymphatics. Nat Med.

