# Correction: Non-toxic dose of liposomal honokiol suppresses metastasis of hepatocellular carcinoma through destabilizing EGFR and inhibiting the downstream pathways

**DOI:** 10.18632/oncotarget.27369

**Published:** 2020-09-01

**Authors:** Jianhong Yang, Heying Pei, Hong Luo, Afu Fu, Hansuo Yang, Jia Hu, Chengjian Zhao, LuLu Chai, Xiang Chen, Ximing Shao, Chunyu Wang, Wenshuang Wu, Li Wan, Haoyu Ye, Qiang Qiu, Aihua Peng, Yuquan Wei, Li Yang, Lijuan Chen

**Affiliations:** ^1^ State Key Laboratory of Biotherapy and Cancer Center, West China Hospital, Sichuan University, and Collaborative Innovation Center for Biotherapy, Chengdu, P.R. China; ^2^ Department of Ultrasonic Medicine, West China Second Hospital, Sichuan University, Chengdu, China; ^3^ School of Pharmacy, Chengdu University of TCM, The Ministry of Education Key Laboratory of Standardization of Chinese Herbal Medicine, State Key Laboratory Breeding Base of Systematic Research, Development and Utilization of Chinese Medicine Resources, Chengdu, China

**This article has been corrected:** Due to errors during data processing, accidental duplication occurred between the “Con” and “LH” images of LO2 group in Fig.1D. The percentage of Q4 of the “Con” was wrongly labeled as “14.3%”; the correct value is “4.3%”. The corrected Figure 1 is shown below. The authors declare that these corrections do not change the results or conclusions of this paper.

Original article: Oncotarget. 2017; 8:915–932. 915-932. https://doi.org/10.18632/oncotarget.13687

**Figure 1:**
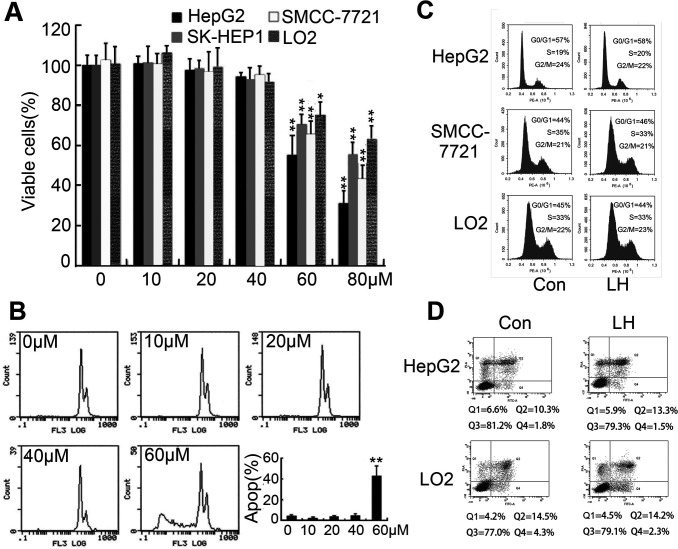
Determination of non-toxic concentration of LH.

(**A**) The inhibitory effect of LH on HepG2, SM7721, SK-HEP1 and LO2 cell proliferation. The inhibition of cell proliferation was determined using MTT assay. The cells were treated with empty liposome or the indicated concentrations of LH for 24 h. Data represent the mean ± standard error (SE) from three independent experiments. *P < 0.05, **P < 0.01,compared with the empty liposome group. (**B**) HepG2 Cells were treated with empty liposome or different concentrations of LH for 24 h, then collected, stained with PI, and analyzed by flow cytometry for apoptosis. Data represent the mean ± SE from three independent experiments. **P < 0.01,compared with the empty liposome group. (**C**) Cells (HepG2, SMCC7721 and LO2) were treated with empty liposome or 40 μM LH for 24 h, then collected, stained with PI, and analyzed by flow cytometry for cell cycle. (**D**) Cells (HepG2 and LO2) were treated with empty liposome or 40 μM LH for 24 h, then collected, stained with PI and AnnexinV, and analyzed by flow cytometry for apoptosis.

